# Chirality-Induced Spin Selectivity (CISS) Effect: Magnetocurrent–Voltage Characteristics with Coulomb Interactions I

**DOI:** 10.1021/acs.jpcc.2c08807

**Published:** 2023-04-03

**Authors:** Karssien Hero Huisman, Jan-Brian Mi-Yu Heinisch, Joseph Marie Thijssen

**Affiliations:** Kavli Institute of Nanoscience, Delft University of Technology, 2628 CJ Delft, The Netherlands

## Abstract

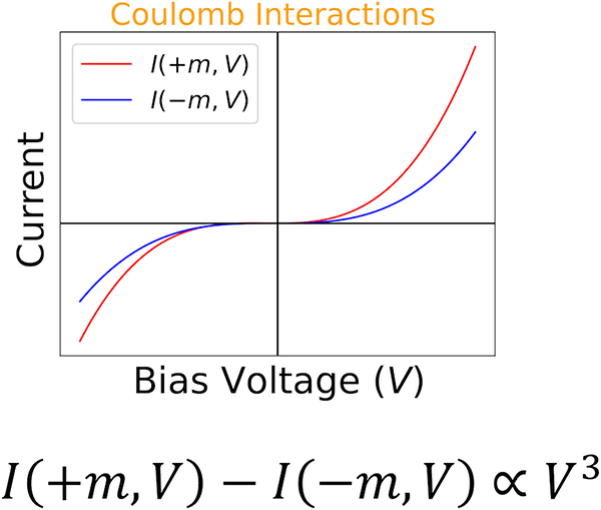

One of the manifestations of chirality-induced spin selectivity (CISS) is the appearance of a magnetocurrent. Magnetocurrent is the observation that the charge currents at finite bias in a two terminal device for opposite magnetizations of one of the leads differ. Magnetocurrents can only occur in the presence of interactions of the electrons either with vibrational modes or among themselves through the Coulomb interaction. In experiments on chiral molecules assembled in monolayers, the magnetocurrent seems to be dominantly cubic (odd) in bias voltage while theory finds a dominantly even bias voltage dependence. Thus far, theoretical work has predicted a magnetocurrent which is even bias. Here we analyze the bias voltage dependence of the magnetocurrent numerically and analytically involving the spin–orbit and Coulomb interactions (through the Hartree–Fock and Hubbard One approximations). For both approximations it is found that for strong Coulomb interactions the magnetocurrent is dominantly odd in bias voltage, confirming the symmetry observed in experiment.

## 1 Introduction

Chirality-induced spin selectivity (CISS) is a term that classifies a collection of experimental observations on chiral molecules. These observations were made in photoemission,^1−4^ Hall-type,^5,6^ and transport experiments^7−15^ (for an extensive overview, see ref (16)). Photoemission experiments show that a layer of chiral molecules has a different transmission probability for spin up and down electrons, i.e., the transmission probability is spin dependent. Hall-type experiments show that self-assembled monolayers of chiral molecules magnetize when placed on a substrate. This magnetization changes with the chirality of the molecules,^5^ and it decreases over time.^6^ In two terminal transport experiments, CISS manifests itself as the appearance of magnetocurrent (MC). MC is the observation that the currents for non-zero bias differ for opposite magnetizations of the lead. Theory initially has mostly focused on the spin dependence of the transmission. The spin–orbit coupling of the molecule’s constituents in combination with the chirality of the molecule induce an asymmetry in the transmission probability for spin up and down electrons of the order 10^–5^% when no decoherence is considered.^17^ It has been shown that the chirality of the molecule in combination with the spin–orbit coupling of the substrate can induce an asymmetry in the transmission probability for spin up and down electrons of a few percent,^18,19^ consistent with theoretical work on^20,21^ and findings in photoemission experiments. A spin dependent transmission does however not imply a MC.^19,22,23^

MC simply is not possible in a fully coherent, noninteracting particle picture according to Büttiker’s reciprocity theorem for two terminal systems^24^ so that modeling beyond this simplified picture is necessary.^22^

Thus, in addition to the chirality of the molecule and spin–orbit coupling, interactions need to be present^25^ to obtain a magnetocurrent; in other words, interactions are a necessary ingredient for translating a spin dependent transmission into a non-zero MC. Several authors have made attempts to reveal MC in a theoretical description in chiral structures by including electron–phonon interactions,^26^ electron–electron interactions,^27^ or a generic decoherence probe.^19,23^ Some authors^28,29^ have proposed alternative explanations based on chirality-induced interface magnetization. In describing two terminal transport measurements of the CISS effect there are three points that need to be addressed by theory. The first point is the Onsager–Casimir reciprocity, which states that the *linear* conductance terms at equilibrium are equal for opposite magnetizations: *G*_1_(*m*) = *G*_1_(−*m*).^22,30−32^ Deviations from Onsager–Casimir reciprocity are not expected and theories should therefore reproduce this or provide a strong reason for their violation.^33^ The second point is the odd/even behavior of the magnetocurrent Δ*I* in bias voltage. From Onsager–Casimir reciprocity, it follows that the magnetocurrent Δ*I* is nonlinear in bias voltage.^19,23^ Experiments^8−15^ find that the MC is dominantly odd in bias voltage indicating that a cubic dependence dominates (Δ*I* ∝ *V*^3^).

The theoretical work of Yang, van der Wal, and van Wees^23^ modeled interactions with the vibrational modes via an extra node which is placed between the molecule and one of the leads, forcing the electrons to move through this node and thereby fully decohere. They found that the magnetocurrent is dominantly even in bias voltage (Δ*I* ∝ *V*^2^). An analysis from our group that modeled interactions with the vibrational modes via the Büttiker voltage probe method and realistic parameters for the electronic structure came to the same conclusion.^19^ Theoretical work of refs (25 and 34) for mesoscopic metallic samples (quantum Hall bar, chaotic cavity) finds that Δ*I* is dominantly even in bias voltage. In ref (35), Δ*I* is odd, but it seems to violate Onsager–Casimir reciprocity since Δ*I* is linear in bias voltage. The discrepancy between the odd Δ*I*–*V* characteristics of the experiment and the even ones of the theory remains a puzzling problem and it is this discrepancy which is the main topic of this work. The third point entails the size of the effect. In experiments, it can reach values of 1–80%, while from theoretical model calculations for realistic parameters,^19,36^ values of less than 1% have been reported. Fransson^27^ found that for Coulomb interactions in the Hubbard One approximation the size of the CISS effect can reach values on the order 10%. In this study, we will consider Coulomb interactions using the Hartree–Fock approximation (HFA) and the Hubbard One approximation (HIA) for more or less realistic parameters, focusing on the bias voltage dependence of the magnetocurrent. The article is structured as follows: in section 2, we describe the scattering region; in section 3, we present our numerical results; in section 4, we give an explanation of our results; and we present our main conclusions in section 5.

## 2 Model Description

The Hamiltonian of a molecular transport junction is given by
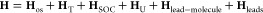
1where **H**_os_ is the on-site Hamiltonian, **H**_T_ is the hopping Hamiltonian, **H**_SOC_ is the hopping Hamiltonian due to spin–orbit coupling, **H**_U_ describes the Coulomb interactions, **H**_lead–molecule_ describes the coupling of the molecule to the leads, and **H**_leads_ describes the Hamiltonian of the leads. The on-site Hamiltonian is given by **H**_os_ = ∑_*k*_ϵ_*k*_*n̂*_*k*_, the on-site energy will be set to zero (ϵ_*k*_ = 0) throughout this paper. The hopping Hamiltonian is given by **H**_T_ = −∑_*k*_*tĉ*_*k*+1_^†^*ĉ*_*k*_ + h.c., where *t* is the hopping parameter and h.c. denotes the Hermitian conjugate. In this work, the sites are arranged in a helix with radius *a* and pitch *c*. *N* is the number of sites within one winding and *M* is the number of windings such that *MN* is the total number of sites in the molecule. For the hopping Hamiltonian due to spin–orbit coupling, we use the model of Fransson^27^ which couples next-nearest neighbors **H**_SOC_ = ∑_*k*_λ(*iv⃗*_*k*_·σ⃗)*ĉ*_*k*+2_^†^*ĉ*_*k*_ + h.c., where λ is the spin–orbit coupling parameter, the components of σ⃗ are the Pauli matrices, *v⃗*_*k*_ = *d⃗*_*k*+1_ × *d⃗*_*k*+2_ and *d⃗*_*k*+*n*_ = (*r⃗*_*k*_ – *r⃗*_*k*+*n*_)/|*r⃗*_*k*_ – *r⃗*_*k*+*n*_|, with *r⃗*_*k*_ the coordinates of site *k* on a helix:
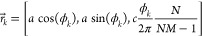
and ϕ_*k*_ = 2π(*k* – 1)/*N*, *k* ∈ *MN*. We take *M* = 1, *N* = 8 and *a* = 1, *c* = 1. Note that due to the spin-dependent hopping term the lattice is non-bipartite. **H**_U_ contains the Coulomb interactions, we take those to be on-site:
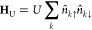
2with *U* the Coulomb interaction strength. We model the leads using the wide-band limit, meaning that the self-energies are purely imaginary and independent of energy. The diagonal matrix elements of lead α that are coupled to the molecule are given by γ(**1** + *p*_*z*_^α^σ_*z*_) and are zero otherwise. Here γ is the coupling strength and *p*_*z*_^α^ ∈ [−1, 1] is the magnetic polarization of lead α. For the right lead, we take *p*_*z*_^R^ = 0, and we couple the left lead to the two leftmost sites and the right lead to the two right most sites. We aim at using realistic parameters corresponding to a molecule consisting of carbon atoms. We take the hopping parameter *t* = 2.4 eV.^37^ Due to the image-charge effect,^38^ the effective on-site Coulomb interaction of carbon, which for an isolated molecule is *U*_C_ = 10.06 eV,^39^ will be lowered to an extent which sensitively depends on the molecule–lead separation. To investigate the effect of *U* on the bias dependence of the magnetocurrent, we vary *U* to a maximum value of 4.8 eV. The spin–orbit coupling parameter of helicene is λ = 6 meV^17^ (therefore, λ/*t* ≈ 10^–3^). To also investigate its effect on the bias dependence of the magnetocurrent, we will vary λ between 10^–3^*t* and 10^–1^*t*. Furthermore, we take *T* = 300 K, the coupling strength the lead is taken as γ = 0.5 eV^40^ and *p*_*z*_^L^ = 0.5. The Coulomb interactions cause a shift in the on-site energies of the Hamiltonian of *U*/2 causing the molecular spectrum to be symmetric around *U*/2 for bipartite lattices. At zero bias voltage the chemical potentials of the left and right lead are given by the Fermi energy *E*_F_. In that case, for , the molecule is charge neutral and *E*_F_ lies precisely between the highest occupied molecular orbital (HOMO) and lowest unoccupied molecular orbital (LUMO) energy. However, in molecular junctions, the molecule is rarely charge neutral due to charge transfer, which corresponds to  (lying closer to either the HOMO or LUMO energy). Therefore, we also vary the Fermi energy between the energy of the HOMO and LUMO level. In appendix A, we state the retarded and advanced two-point Green’s functions in the Hartree–Fock and the Hubbard One approximation derived with the equation of motion technique (analogous to chapter 12 of ref (41)). These Green’s functions are expressed in terms of the average electron densities for site *k* with spin *s*, ⟨*n*_*ks*_⟩, which we determine self-consistently from the Green’s functions; see eq (eq 11). Every iteration *m* has an input and an output electron density, and as convergence criterion for the *m*th iteration, we use: |⟨*n*_*ks*_^in,*m*^⟩ – ⟨*n*_*ks*_^out,*m*^⟩| < 10^–5^. The Hamiltonian without interactions (*U* = 0) is defined as **H**_0_ = **H**_os_ + **H**_T_ + **H**_SOC_ and is constructed with the Kwant code^42^ and the Qsymm code.^43^ We have implemented a nonequilibrium transport code which can be found in https://github.com/khhuisman/CISS_CoulombInteraction. In this code, we determine the electron density as follows. Suppose we want to calculate the electron density for the decreasing or increasing bias voltages {0, *V*_1_, *V*_2_, ...}, (|*V*_*i*+1_| > |*V*_*i*_|). First of all, we start our self-consistent calculation at zero bias voltage where we expect that every site is approximately half filled; therefore we take this as an initial guess . Then we self-consistently determine the electron densities for *V* = 0 and obtain the converged result ⟨*n*_*ks*_^converged^(*V* = 0)⟩. We then use these values as an initial guess for the next bias voltage *V*_1_: ⟨*n*_*ks*_^in,*m*=0^(*V* = *V*_1_)⟩ = ⟨*n*_*ks*_^converged^(*V* = 0)⟩. We always use the output of a self-consistent calculation as initial guess for the next bias voltage: ⟨*n*_*ks*_^in,*m*=0^(*V* = *V*_*i*+1_)⟩ = ⟨*n*_*ks*_^converged^(*V* = *V*_*i*_)⟩ to adiabatically connect the two solutions. This procedure is done separately for positive and negative bias, and both times we start in *V* = 0. Furthermore, we employ linear mixing of the electron densities meaning that the input for iteration *m* + 1 is a linear combination of the input and output of iteration *m*: ⟨*n*_*ks*_^in,*m*+1^(*V*)⟩ = (1 – α)⟨*n*_*ks*_^out,*m*^(*V*)⟩ + α⟨*n*_*ks*_^in,*m*^(*V*)⟩ characterized by the parameter α ∈ [0, 1). The used values of α vary between the different Coulomb interaction strengths and are indicated in the code.

## 3 Results

We now turn to a two terminal system with Coulomb interactions. The transmission then depends on the bias voltage *V* through the Coulomb potential, and in the HFA and HIA, this is expressed in terms of the electron densities, that depend on the bias voltage applied to the molecule. This means that the transmission becomes voltage dependent: *T*_LR_(*m*) → *T*_LR_(*m*, *V*) = *T*_LR_(*m*, ⟨*n*_1*↑*_(*m*, *V*)⟩, ⟨*n*_1*↓*_(*m*, *V*)⟩, ..., ⟨*n*_*ks*_(*m*, *V*)⟩). The current into the left lead is then given by
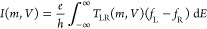
3where *f*_α_ = *f*(*E*, μ_α_, β) is the Fermi–Dirac distribution of the lead α with chemical potential μ_α_ at  with *T* the temperature of the lead. The transmission is given by *T*_LR_ = Tr[**Γ**_L_**G**^+^**Γ**_R_**G**^–^] with **G**^+^ the retarded Green’s function. Assuming symmetric capacitive coupling to the left and right lead, the chemical potentials of the left and right leads are  and , respectively, with *E*_F_ the Fermi energy and *V* the bias voltage. Using eq 3, we can write the magnetocurrent as
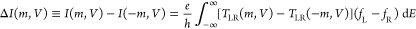
4

**Figure 1:**
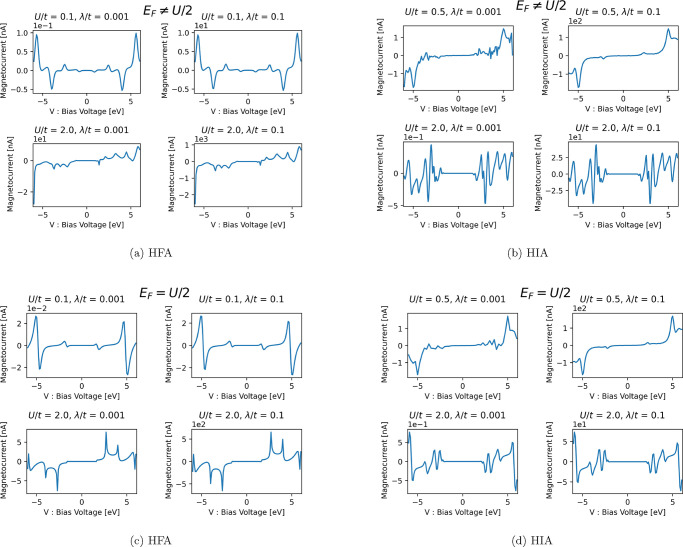
Δ*I*(*m*, *V*) for the helical geometry. Asymmetric chemical potential (a) in the HFA, (b) in the HIA. Symmetric chemical potential (c) in the HFA, (d) in the HIA.

In Figure 1, Δ*I*(*m*, *V*) is plotted as a function of bias voltage in the HFA and HIA respectively. In Figure 1a, the magnetocurrent is plotted for  in the HFA, and we see that for small *U*/*t* (upper panels) the magnetocurrent is dominantly even in bias voltage and for large *U*/*t* (lower panels) the magnetocurrent is dominantly odd in bias voltage for . In Figure 1b, the magnetocurrent is plotted for  in the HIA, and we see that it is a dominantly odd function of the bias voltage. In Figure 1c,d, we see that Δ*I*(*m*, *V*) is dominantly odd in voltage for . From the numerical results, it is clear that Δ*I*(*m*, *V*) is dominantly odd in bias voltage in both the HFA and HIA for most cases, the only exception being  for small *U*/*t* < 1 in the HFA for which the magnetocurrent is dominantly even. Here we simply stated our numerical results, in section 4 we present a theoretical analyses to explain them. Now follow some other results, our calculation shows that in both the HFA and HIA the polarization of the current  is less than 1% even if relatively large values of the spin–orbit coupling parameter are used. Also it is found that when the molecule changes its chirality, Δ*I*(*m*, *V*) exactly changes sign Δ*I*(*m*, *V*) → −Δ*I*(*m*, *V*). Furthermore, if the spin–orbit coupling parameter is set to zero, the magnetocurrent vanishes.

## 4 Discussion

In this section, we provide theoretical arguments to qualitatively explain our numerical results. We can expand the magnetocurrent (eq 4) in bias voltage as Δ*I*(*m*, *V*) = Δ*G*_1_(*m*)*V* + Δ*G*_2_(*m*)*V*^2^ + Δ*G*_3_(*m*)*V*^3^ + ..., where
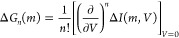
5Onsager–Casimir reciprocity,^30,31^ implies that Δ*G*_1_(*m*) = 0. To show that this relates back to time-reversal symmetry at equilibrium (*V* = 0 and equal temperatures for both leads^44^), we write Δ*G*_1_(*m*) in terms of the transmission:
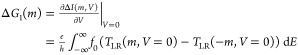
6

Here we adopted the notation *f*_0_^′^ = −∂_E_*f*(*E*, *E*_F_, β). Onsager requires that in equilibrium the system is time-reversal symmetric. This in combination with current conservation implies for the transmission that *T*_LR_(*m*, *V* = 0) = *T*_LR_(−*m*, *V* = 0) so that indeed Δ*G*_1_(*m*) = 0. As a consequence of this, the magnetocurrent can only be nonlinear in bias voltage Δ*I*(*m*, *V*) ∝ Δ*G*_2_(*m*)*V*^2^ + Δ*G*_3_(*m*)*V*^3^ + ... in accordance with the conclusions of refs (22 and 23). From our numerical calculations we indeed verify that the transmission satisfies *T*_LR_(*m*, *V* = 0) = *T*_LR_(−*m*, *V* = 0) in the HFA and HIA; therefore Onsager–Casimir reciprocity is satisfied. The nonlinear coefficients Δ*G*_2_(*m*), Δ*G*_3_(*m*), ... may however vanish. To show that Δ*G*_2_(*m*) is finite, we analyze it via eq 5. At equilibrium, TRS implies that the occupation difference Δ*n*_*ks*_(*m*, *V*) = ⟨*n*_*ks*_(*m*, *V*)⟩ – ⟨*n*_*ks̅*_(−*m*, *V*)⟩ (*s̅* denotes that we flip spin *s*) is zero Δ*n*_*ks*_(*m*, *V* = 0) = 0. At nonzero bias, deviation from equilibrium manifests itself through Δ*n*_*ks*_(*m*, *V*) which no longer vanishes when the bias voltage is finite. Therefore, from eq 5 (SI section 1.1.2), we have
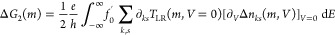
7where ∂_*ks*_ = ∂_⟨*n*_*ks*_(*m*,*V*=0)⟩_. Equation 7 can be understood as follows: the Green’s function is time-reversal symmetric except for the electron densities. As the zeroth order term Δ*n*_*ks*_(*m*, *V* = 0) = 0, Δ*G*_2_(*m*) can only scale with the first order derivative of Δ*n*_*ks*_(*m*, *V*) at *V* = 0. Intuitively this makes sense: to what extent TRS is broken out of equilibrium (*V* ≠ 0) scales with the occupation difference: Δ*n*_*ks*_(*m*, *V*) = ⟨*n*_*ks*_(*m*, *V*)⟩ – ⟨*n*_*ks̅*_(−*m*, *V*)⟩. Deviations from TRS thus manifest themselves through Δ*n*_*ks*_(*m*, *V*). If this deviates from zero, Δ*G*_2_(*m*) will too. If TRS is present out of equilibrium for every voltage (⟨*n*_*ks*_(*m*, *V*)⟩ = ⟨*n*_*ks̅*_(−*m*, *V*)⟩) then Δ*G*_2_(*m*) ∝ ∂_*V*_Δ*n*_*ks*_(*m*, *V* = 0) = 0 and there is no magnetocurrent as expected due to Büttiker’s reciprocity theorem for two terminal systems.^24^ Furthermore, if *U* = 0, then Δ*I*(*m*, *V*) = 0, since every derivative with respect to bias *V* yields an electron density multiplied by *U*. This shows the importance of going beyond the noninteracting particle picture. From eq 5 (SI section 1.1.3), we have for Δ*G*_3_(*m*):
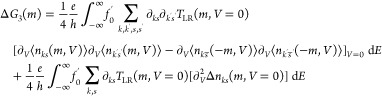
8for a symmetrically biased junction. For small *V*, we expect that Δ*n*_*ks*_(*m*, *V*) varies linearly with bias voltage *V*. It can then be shown that Δ*G*_3_(*m*)/Δ*G*_2_(*m*) ∝ *U* in the HFA and HIA (SI section 1). This is consistent with Figure 1a since Δ*I*(*m*, *V*) changes from even to odd with increasing *U*, and we see that for large *U*, the cubic term in the magnetocurrent tends to dominate. In the HIA we see similar behavior only for much smaller *U* than considered in Figure 1b. The numerical results for Figure 1a,b shows that the even/odd behavior of Δ*I*(*m*, *V*) can change much compared to . At  and *V* = 0, the system is not exactly half-filled which is more the rule than the exception in molecular junctions due to charge transfer to the molecule. Due to the large Coulomb interactions this will result in a magnetocurrent which is odd in bias voltage.

## 5 Conclusion

In this work, we studied the voltage dependence of the magnetocurrent for a system with Coulomb interactions (in the HFA and HIA). The system we studied has next-nearest neighbor, spin-dependent hopping that causes the lattice to be non-bipartite. Our numerical results show that the magnetocurrent is odd in bias voltage in both the HFA and HIA for strong Coulomb interactions (*U* > *t*) in agreement with experiments.^8−15^ Furthermore, we verified that the Onsager–Casimir reciprocity is satisfied, as expected. For a large spin–orbit coupling parameter (λ/*t* = 0.1), we found that the size of the effect is on the order of 0.1% which is of the same order as our previous work on Büttiker voltage probes.^19^ How a bipartite lattice with spin–orbit coupling affects the voltage dependence of the magnetocurrent will be considered in a separate work.

## Appendix A: Electron Green’s Function

A derivation of the Green’s functions is done in Chapter 12 of Haug and Jauho.^41^ In this section, we simply give the Green’s functions for a system with spin–orbit coupling. The Hamiltonian without interactions (*U* = 0) is defined as **H**_0_ = **H**_os_ + **H**_T_ + **H**_SOC_. The retarded Green’s function in the Hartree–Fock approximation is given by

9

and the retarded Green’s function in the Hubbard One approximation is given by

10

where **Σ** is the retarded self-energy which in the wide band limit for a magnetized left lead is given by

and **n** = ∑_*ks*_⟨*n*_*ks̅*_⟩*n̂*_*ks*_ is a diagonal matrix with the electron densities on the diagonal. For these Green’s functions, the density of states are symmetric around the Fermi energy  if the lattice is bipartite. The electron density for site *k* with spin *s* is given by

11

In eq 9, the term ∑_*ks*_⟨*n*_*ks̅*_⟩*n̂*_*ks*_ is the result of a Wick contraction of the term *n*_*k↑*_*n*_*k↓*_ in eq 2. This contraction in principle also allows for the term^45,46^ −⟨*c*_*k*↑_^†^*c*_*k*↓_⟩*c*_*k*↓_^†^*c*_*k*↑_ + h.c., which we will call the noncollinear Hubbard model. In that case, we obtain (SI, section 2) the following noncollinear (NC) Hartree–Fock Green’s function:

12

and the the noncollinear Hubbard One Green’s function:

13

where we defined **ρ** = ∑_*ks*_⟨*c*_*ks̅*_^†^*c*_*ks*_⟩*c*_*ks*_^†^*c*_*ks̅*_. The expectation value ⟨*c*_*ks̅*_^†^*c*_*ks*_⟩ vanishes in the absence of spin–orbit coupling, and it is expected that it does not change the result of our calculations. Indeed, when we include this term in our calculations the bias dependence of the magnetocurrent does not change with respect to ⟨*c*_*ks̅*_^†^*c*_*ks*_⟩ = 0 for all of the considered values of the spin–orbit coupling, Coulomb interaction strength, and Fermi energy. Only in the HFA for , , and  the size of the effect increases significantly to a few percent for low bias voltage. However, this increase in the size of the effect happens for a particular choice Fermi energy, and it is not clear to us why that happens; however, we suspect this is due to a numeric instability. Furthermore, for large Coulomb interactions  the noncollinear Hubbard One Green’s function (eq 13) gives more accurate results, and the size of the effect remains less than 1% while the magnetocurrent remains odd in bias voltage.

## Supporting Information Available

The Supporting Information contains: The Supporting Information is available free of charge at https://pubs.acs.org/doi/10.1021/acs.jpcc.2c08807.

Coulomb Interactions: Higher Order Differential Conductance Coefficients. Derivation of Electron Green’s Function. Evenness or Oddness of Magnetocurrent (PDF)
