# Supplementary material for: Chirality-Induced Spin Selectivity (CISS) Effect: Magnetocurrent–Voltage Characteristics with Coulomb Interactions I
Source: J Phys Chem C Nanomater Interfaces. 2023 Apr 3;127(14):6900–5. doi: 10.1021/acs.jpcc.2c08807 (PMC10108364; doi:10.1021/acs.jpcc.2c08807)
Supplement: Supplementary file 1 — jp2c08807_si_001.pdf [file jp2c08807_si_001.pdf]

# CISS Effect: Magnetocurrent-Voltage Characteristics with Coulomb Interactions I

## Authorship


- Karssien Hero Huisman*,
  - *email*: k.h.huisman@tudelft.nl
  - *affiliation*: Kavli Institute of Nanoscience, Delft University of Technology, 2628 CJ Delft, The Netherlands
  - *telephone*: +31651964048
- Jan-Brian Mi-Yu Heinisch,
  - *email*: j.m.heinisch@student.tudelft.nl
  - *affiliation*: Kavli Institute of Nanoscience, Delft University of Technology, 2628 CJ Delft, The Netherlands,
- Joseph Marie Thijssen,
  - *email*: j.m.thijssen@tudelft.nl,
  - *affiliation*: Kavli Institute of Nanoscience, Delft University of Technology, 2628 CJ Delft, The Netherlands,
  - *telephone*: +31(0)152788457

# Supporting Information

# Contents


**1 Coulomb Interactions: Higher Order Differential Conductance Coefficients** **2**

1.1 Hartree-Fock: Differential conductance Coefficients . . . . . 2

1.1.1 Onsager-Casimir Reciprocity . . . . . 2

1.1.2 Coefficient $\Delta G_2(m)$ . . . . . 3

1.1.3 Coefficient $\Delta G_3(m)$ . . . . . 3

1.2 Comparing non-linear coefficients . . . . . 3

1.3 Transmission derivatives . . . . . 4

1.3.1 Hartree Fock . . . . . 4

1.3.2 Hubbard One . . . . . 5

1.4 Unitarity of the transmission derivatives . . . . . 5

**2 Derivation of Electron Green's Function** **6**

2.1 Definitions . . . . . 6

2.2 Green's Function . . . . . 6

2.3 Equation of Motion Technique . . . . . 7

2.4 Coupled, Interacting System . . . . . 7

2.5 Retarded Green's Function: Equation of Motion . . . . . 8

2.6 Hartree - Fock Approximation . . . . . 9

2.7 Hubbard One Approximation . . . . . 10

**3 Evenness or Oddness of Magnetocurrent** **14**

**4 Bibliography** **16**

# 1 Coulomb Interactions: Higher Order Differential Conductance Coefficients

In section 1.1 the coefficients $\Delta G_2(m), \Delta G_3(m)$ will be calculated via equation (eq.) (5) from the main text and it will be shown that Onsager-Casimir reciprocity ($\Delta G_1(m) = 0$) follows from time-reversal symmetry in equilibrium. In sec. 1.2 we compare $\Delta G_2(m), \Delta G_3(m)$ for a system with Coulomb interactions in the Hartree-Fock approximation that satsifies Onsager-Casimir reciprocity.

## 1.1 Hartree-Fock: Differential conductance Coefficients

Let us introduce the notation:

$$f_\alpha(E) = [\exp(\beta_\alpha(E-\mu_\alpha))+1]^{-1} \tag{S.1}$$

$$f'_\alpha \equiv \partial_{\mu_\alpha} f_\alpha, \quad f''_\alpha \equiv \partial^2_{\mu_\alpha} f_\alpha \tag{S.2}$$

Where $\mu_\alpha$ is the the chemical potential of lead $\alpha$ and $\beta_\alpha = \frac{1}{k_B T_\alpha}$ with $T_\alpha$ the temperature of lead $\alpha$. We will only consider equal temperatures for the leads $\beta_{\mathrm{L,R}} = \frac{1}{k_B T}$. The chemical potential of the left, right lead read given by:

$$\mu_L = E_{\mathrm{F}} + \eta V, \quad \mu_R = E_{\mathrm{F}} - (1-\eta)V$$
$$\partial_V \mu_L = \eta, \quad \partial_V \mu_R = -(1-\eta) \tag{S.3}$$

Here $\eta$ is the capacitive coupling. We define $f_0 = f_{L,R}(E)|_{V=0} = [\exp(\beta(E-E_{\mathrm{F}}))+1]^{-1}$ and $f_0^{'} = \partial_{\mu_{L,R}} f_{L,R}(E)|_{V=0}$.

### 1.1.1 Onsager-Casimir Reciprocity

Onsager demands that in equilibrium the system is time-reversal symmetric. To be unambiguous we define "equilibrium" as $V = 0$ (i.e. $\mu_L = \mu_R$) and equal temperatures for both leads (i.e. $T_L = T_R$) if one of these conditions is violated we say the system is "out of equilibrium".[1] For the transmission time-reversal symmetry implies that $T_{\mathrm{LR}}(m, V=0) = T_{\mathrm{RL}}(-m, V=0)$. Also we have $T_{\mathrm{RL}}(m, V) = T_{\mathrm{LR}}(m, V)$, due to current conservation $I_L + I_R = 0$. Combining time-reversal symmetry and current conservation this implies for the transmission that:

$$T_{\mathrm{LR}}(m, V=0) = T_{\mathrm{LR}}(-m, V=0) \tag{S.4}$$

This implies that $\Delta G_1(m) = 0$ see eq. (6) in the main text.

### 1.1.2 Coefficient $\Delta G_2(m)$

From eq. (5) in the main text we obtain the following expression for $\Delta G_2(m)$ (invoking eq. (S.4)):

$$
\begin{aligned}
\Delta G_2(m) &= \frac{1}{2}\frac{\partial \Delta I(m, V=0)}{\partial V^2} \\
&= \frac{1}{2}\frac{e}{h}\int_{-\infty}^{\infty}\Big[\partial_V\, T_{\mathrm{LR}}(m, V=0) - \partial_V\, T_{\mathrm{LR}}(-m, V=0)\Big] f_0' dE \\
&= \frac{1}{2}\frac{e}{h}\int_{-\infty}^{\infty}\sum_{k,s}\partial_{ks}\, T_{\mathrm{LR}}(m, V=0)\Big[\partial_V \Delta n_{ks}(m, V=0)\Big] f_0' dE
\end{aligned}
\tag{S.5}
$$

Here we adopted the notation $\partial_{\langle n_{ks}(m,V=0)\rangle} \equiv \partial_{ks}$ and in the last line $\Delta n_{ks}(m, V) \equiv \langle n_{ks}(m, V)\rangle - \langle n_{k\bar{s}}(-m, V)\rangle$. We used that $\partial_{\langle n_{ks}(m,V=0)\rangle}\, T_{\mathrm{LR}}(m, V=0) = \partial_{\langle n_{k\bar{s}}(-m,V=0)\rangle}\, T_{\mathrm{LR}}(-m, V=0)$ as we will show in sec. 1.4.

### 1.1.3 Coefficient $\Delta G_3(m)$

From eq. (5) in the main text we obtain the following expression for $\Delta G_3(m)$ (invoking eq. (S.4)):

$$
\begin{aligned}
\Delta G_3(m) &= \frac{1}{6}\frac{\partial^3 \Delta I(m, V=0)}{\partial V^3} \\
&= \frac{1}{4}\frac{e}{h}\int_{-\infty}^{\infty} f_0''(2\eta-1)\sum_{k,s}\partial_{ks}\, T_{\mathrm{LR}}(m, V=0)\Big[\partial_V \Delta n_{ks}(m, V=0)\Big] \\
&+ \frac{1}{4}\frac{e}{h}\int_{-\infty}^{\infty} f_0' \sum_{k,k',s,s'}\partial_{ks}\partial_{k's'}\, T_{\mathrm{LR}}(m, V=0)\Big[\partial_V\langle n_{ks}(m,V)\rangle\partial_V\langle n_{k's'}(m,V)\rangle - \partial_V\langle n_{k\bar{s}}(-m,V)\rangle\partial_V\langle n_{k'\bar{s}'}(-m,V)\rangle\Big]_{V=0} dE \\
&+ \frac{1}{4}\frac{e}{h}\int_{-\infty}^{\infty} f_0' \sum_{k,s}\partial_{ks}\, T_{\mathrm{LR}}(m, V=0)\Big[\partial_V^2 \Delta n_{ks}(m, V=0)\Big] dE
\end{aligned}
\tag{S.6}
$$

To simplify the expression we used $\partial_{\langle n_{ks}(m,V=0)\rangle}\partial_{\langle n_{k's'}(m,V=0)\rangle}\, T_{\mathrm{LR}}(m, V=0) = \partial_{\langle n_{k\bar{s}}(-m,V=0)\rangle}\partial_{\langle n_{k'\bar{s}'}(-m,V=0)\rangle}\, T_{\mathrm{LR}}(-m, V=0)$ and $\partial_{\langle n_{ks}(m,V=0)\rangle}\, T_{\mathrm{LR}}(m, V=0) = \partial_{\langle n_{k\bar{s}}(-m,V=0)\rangle}\, T_{\mathrm{LR}}(-m, V=0)$. The validity of these equations is demonstrated in section 1.4.

## 1.2 Comparing non-linear coefficients

In section 1.3 we show that: $\partial_{ks}\partial_{k's'}\, T_{\mathrm{LR}}(m, V=0) = U^2 T^{(2)}_{ks,k's'}(m) \propto U^2$ and $\partial_{ks}\, T_{\mathrm{LR}}(m, V=0) = U T^{(2)}_{ks}(m) \propto U$. For small bias we expect $\partial_V\langle n_{ks}(m, V=0)\rangle, \partial_V\langle n_{k\bar{s}}(-m, V=0)\rangle$ to vary approximately linear in bias voltage ($\partial_V^2 n_{ks}(\pm m) \propto 0$). Let us further assume symmetric capacative coupling to the leads $\eta = 1/2$ then the ratio between $\Delta G_3(m)$ and $\Delta G_2(m)$ is given by;

$$\Delta G_3(m)/\Delta G_2(m) \tag{S.7}$$

$$= \frac{1}{2}\frac{\int_{-\infty}^{\infty} f_0' \sum_{k,k',s,s'} \partial_{ks}\partial_{k's'} T_{\rm LR}(m,V=0)\Big[\partial_V\langle n_{ks}(m,V)\rangle\partial_V\langle n_{k's'}(m,V)\rangle - \partial_V\langle n_{k\bar{s}}(-m,V)\rangle\partial_V\langle n_{k'\bar{s}'}(-m,V)\rangle\Big]_{V=0} dE}{\int_{-\infty}^{\infty}\sum_{k,s}\partial_{ks} T_{\rm LR}(m,V=0)\Big[\partial_V\langle n_{ks}(m,V=0)\rangle - \partial_V\langle n_{k\bar{s}}(-m,V=0)\rangle\Big] f_0' dE}$$

$$= \frac{U}{2}\frac{\int_{-\infty}^{\infty} f_0' \sum_{k,k',s,s'} T^{(2)}_{ks,k's'}(m,V=0)\Big[\partial_V\langle n_{ks}(m,V)\rangle\partial_V\langle n_{k's'}(m,V)\rangle - \partial_V\langle n_{k\bar{s}}(-m,V)\rangle\partial_V\langle n_{k'\bar{s}'}(-m,V)\rangle\Big]_{V=0} dE}{\int_{-\infty}^{\infty}\sum_{k,s} T^{(1)}_{ks}(m,V=0)\Big[\partial_V\langle n_{ks}(m,V=0)\rangle - \partial_V n_{k\bar{s}}(-m,V=0)\rangle\Big] f_0' dE} \propto U \tag{S.8}$$

## 1.3 Transmission derivatives

In the previous section we encountered the derivatives: $\sum_{k,s}\partial_{ks} T_{\rm LR}(m,V=0)$ and $\sum_{k,k',s',s}\partial_{k's'}\partial_{ks} T_{\rm LR}(m,V=0)$, where we adopted the notation $\partial_{\langle n_{ks}(m,V=0)\rangle} = \partial_{ks}$. Here we investigate how the derivatives of the transmission scale with the interaction strength $U$ in the HFA and HIA.

### 1.3.1 Hartree Fock

We use $\frac{\partial}{\partial x}\mathbf{A}^{-1} = -\mathbf{A}^{-1}\frac{\partial \mathbf{A}}{\partial x}\mathbf{A}^{-1}$ and that in the HFA Green's function (eq. (9), main text ) at $V=0$ is given by: $\mathbf{G}_0^{\pm}(\epsilon,\mathbf{n}(m,V=0),m) = \Big[\mathbf{I}\epsilon - \mathbf{H}_0 - U\mathbf{n}(m,V=0) \pm \frac{i}{2}\mathbf{\Gamma}(m)\Big]^{-1}$. The derivative $\partial_{ks}$ only acts on the electron densities:

$$\partial_{ks}\mathbf{G}_0^{\pm} = U\mathbf{G}_0^{\pm}\mathbf{X}^{ks}\mathbf{G}_0^{\pm} \tag{S.9}$$

Where we define $\mathbf{X}^{ks} = \sum_{k',s'}\frac{\partial\langle n_{k'\bar{s}'}(m,V=0)\rangle}{\partial\langle n_{ks}(m,V=0)\rangle}\hat{n}_{k's'} = \hat{n}_{k\bar{s}}$. This is a diagonal matrix with only one non-zero element i.e. $\mathbf{X}^{k\uparrow} = \mathrm{diag}(0,0,..,1,0,...,0)$ and $\mathbf{X}^{k\downarrow} = \mathrm{diag}(0,0,..,0,1,...,0)$. The first derivative of the transmission then becomes:

$$\partial_{ks} T_{\rm LR}(m,V=0) = U\mathrm{Tr}\Big[\mathbf{X}^{ks}\Big(\mathbf{G}_0^{+}\mathbf{\Gamma}_R\mathbf{G}_0^{-}\mathbf{\Gamma}_L(m)\mathbf{G}_0^{+} + h.c.\Big)\Big] = UT^{(1)}_{ks}(m,V=0) \propto U \tag{S.10}$$

Now we continue with the second derivative using (S.9):

$$\begin{aligned}
&\partial_{k's'}\partial_{ks} T_{\rm LR}(m,V=0)\\
&= U^2\mathrm{Tr}\Big[\mathbf{X}^{ks}\Big(\mathbf{G}_0^{+}\mathbf{X}^{k's'}\mathbf{G}_0^{+}\mathbf{\Gamma}_R\mathbf{G}_0^{-}\mathbf{\Gamma}_L(m)\mathbf{G}_0^{+} + h.c.\Big)\Big]\\
&+ U^2\mathrm{Tr}\Big[\mathbf{X}^{ks}\Big(\mathbf{G}_0^{+}\mathbf{\Gamma}_R\mathbf{G}_0^{-}\mathbf{X}^{k's'}\mathbf{G}_0^{-}\mathbf{\Gamma}_L(m)\mathbf{G}_0^{+} + h.c.\Big)\Big]\\
&+ U^2\mathrm{Tr}\Big[\mathbf{X}^{ks}\Big(\mathbf{G}_0^{+}\mathbf{\Gamma}_R\mathbf{G}_0^{-}\mathbf{\Gamma}_L(m)\mathbf{G}_0^{+}\mathbf{X}^{k's'}\mathbf{G}_0^{+} + h.c.\Big)\Big] = U^2 T^{(2)}_{k's',ks}(m,V=0) \propto U^2
\end{aligned} \tag{S.11}$$

This thus shows that $\partial_{k's'}\partial_{ks} T_{\rm LR}(m) \propto U^2$.

### 1.3.2 Hubbard One

In the Hubbard One Approximation the Green's function is given by eq. (10). We rewrite it as $\mathbf{G}^{+}_{\text{HIA}} = \mathbf{A}^{-1}\mathbf{B}$ where $\mathbf{A}, \mathbf{B}$ are given by: $\mathbf{A} = \Big[(\epsilon\mathbf{I} - \mathbf{H}_{\text{os}} - U\mathbf{I})(\epsilon\mathbf{I} - \mathbf{H}_0 - \mathbf{\Sigma}) - U\mathbf{n}(\mathbf{H}_{\text{T}} + \mathbf{H}_{\text{SOC}} + \mathbf{\Sigma})\Big]$ and $\mathbf{B} = \epsilon\mathbf{I} - \mathbf{H}_{\text{os}} - U(\mathbf{I} - \mathbf{n})$. Taking the derivative with respect to the electron density of $\mathbf{G}^{+}_{\text{HIA}}$ gives:

$$\partial_{ks}\mathbf{G}^{+}_{\text{HIA}} = U\mathbf{A}^{-1}\Big[\mathbf{X}^{ks}\Big(\mathbf{H}_{\text{T}} + \mathbf{H}_{\text{SOC}} + \mathbf{\Sigma}\Big)\mathbf{G}^{+}_{\text{HIA}} + \mathbf{X}^{ks}\Big] \tag{S.12}$$

$$\begin{aligned}\partial_{k's'}\partial_{ks}\mathbf{G}^{+}_{\text{HIA}} &= U^2\mathbf{A}^{-1}\mathbf{X}^{k's'}\Big(\mathbf{H}_{\text{T}} + \mathbf{H}_{\text{SOC}} + \mathbf{\Sigma})\mathbf{A}^{-1}\Big[\mathbf{X}^{ks}\Big(\mathbf{H}_{\text{T}} + \mathbf{H}_{\text{SOC}} + \mathbf{\Sigma}\Big)\mathbf{G}^{+}_{\text{HIA}} + \mathbf{X}^{ks}\Big] \\ &+ U^2\mathbf{A}^{-1}\mathbf{X}^{ks}\Big(\mathbf{H}_{\text{T}} + \mathbf{H}_{\text{SOC}} + \mathbf{\Sigma}\Big)\mathbf{A}^{-1}\Big[\mathbf{X}^{k's'}\Big(\mathbf{H}_{\text{T}} + \mathbf{H}_{\text{SOC}} + \mathbf{\Sigma}\Big)\mathbf{G}^{+}_{\text{HIA}} + \mathbf{X}^{k's'}\Big]\end{aligned} \tag{S.13}$$

The first derive of the transmission with respect to the electron density is given by:

$$\partial_{ks}T_{\text{LR}}(m, V=0) = \text{Tr}\Big[\mathbf{\Gamma}_L(m)\partial_{ks}\mathbf{G}^{+}_{\text{HIA}}\mathbf{\Gamma}_R\mathbf{G}^{-}_{\text{HIA}} + \mathbf{\Gamma}_L(m)\mathbf{G}^{+}_{\text{HIA}}\mathbf{\Gamma}_R\partial_{ks}\mathbf{G}^{-}_{\text{HIA}}\Big] \propto U \tag{S.14}$$

and with eq. (S.12) we see it is proportional to $U$. The second derivative of the transmission with respect to the electron density is given by:

$$\begin{aligned}\partial_{k's'}\partial_{ks}T_{\text{LR}}(m, V=0) = \text{Tr}\Big[&\mathbf{\Gamma}_L(m)\partial_{k's'}\partial_{ks}\mathbf{G}^{+}_{\text{HIA}}\mathbf{\Gamma}_R\mathbf{G}^{-}_{\text{HIA}} + \mathbf{\Gamma}_L(m)\partial_{ks}\mathbf{G}^{+}_{\text{HIA}}\mathbf{\Gamma}_R\partial_{k's'}\mathbf{G}^{-}_{\text{HIA}} \\ &+ \mathbf{\Gamma}_L(m)\partial_{k's'}\mathbf{G}^{+}_{\text{HIA}}\mathbf{\Gamma}_R\partial_{ks}\mathbf{G}^{-}_{\text{HIA}} + \mathbf{\Gamma}_L(m)\mathbf{G}^{+}_{\text{HIA}}\mathbf{\Gamma}_R\partial_{k's'}\partial_{ks}\mathbf{G}^{-}_{\text{HIA}}\Big] \propto U^2\end{aligned} \tag{S.15}$$

and with eqs. (S.12),(S.13) we see it is proportional to $U^2$.

## 1.4 Unitarity of the transmission derivatives

In obtaining eqs. (S.5) and (S.6) we used the relation $\partial_{\langle n_{ks}(m,V=0)\rangle}T_{\text{LR}}(m, V=0) = \partial_{\langle n_{k\bar{s}}(-m,V=0)\rangle}T_{\text{LR}}(-m, V=0)$ and $\partial_{\langle n_{ks}(m,V=0)\rangle}\partial_{\langle n_{k's'}(m,V=0)\rangle}T_{\text{LR}}(m, V=0) = \partial_{\langle n_{k\bar{s}}(-m,V=0)\rangle}\partial_{\langle n_{k'\bar{s}'}(-m,V=0)\rangle}T_{\text{LR}}(-m, V=0)$ in this section we will show this to be true. We showed that the transmission satisfies $T_{\text{LR}}(m, V=0) = T_{\text{LR}}(-m, V=0)$ in sec 1.1.1. Furthermore TRS at equilibrium implies that that the electron densities satisfy: $\langle n_{ks}(m, V=0)\rangle = \langle n_{k\bar{s}}(-m, V=0)\rangle$. Meaning that the partial derivatives: $\partial_{\langle n_{ks}(m,V=0)\rangle} = \partial_{\langle n_{k\bar{s}}(-m,V=0)\rangle}$. From this it follows that:

$$\begin{aligned}\partial_{\langle n_{ks}(m,V=0)\rangle}T_{\text{LR}}(m, V=0) &= \partial_{\langle n_{k\bar{s}}(-m,V=0)\rangle}T_{\text{LR}}(-m, V=0), \\ \partial_{\langle n_{ks}(m,V=0)\rangle}\partial_{\langle n_{k's'}(m,V=0)\rangle}T_{\text{LR}}(m, V=0) &= \partial_{\langle n_{k\bar{s}}(-m,V=0)\rangle}\partial_{\langle n_{k'\bar{s}'}(-m,V=0)\rangle}T_{\text{LR}}(-m, V=0)\end{aligned} \tag{S.16}$$

Our numerical results satisfy these equations.

## 2 Derivation of Electron Green's Function

In this section the Green's function with Coulomb interactions will be derived in the Hartree-Fock and Hubbard One Approximation. A derivation of these Green's functions is done in the work of Haug and Jauho for a system *without* spin-orbit coupling while the novelty in this section is that we derive these Green's functions for a system *with* spin-orbit coupling.

This section is structured as follows, in 2.1 we will define conventions for our Fourier transforms, in 2.2 we will give a short introduction on Green's function, in 2.3 we will introduce the equation of motion technique, in 2.4 we will define the Hamiltonian, in 2.5 we will derive the equation of motion for the retarded Green's function, in sec. 2.6 we will solve the retarded Green's function in the Hartree-Fock approximation and in sec. 2.7 we will solve the retarded Green's function in the Hubbard One approximation.

### 2.1 Definitions

We define the Fourier transforms conventions:

$$\mathcal{F}(f(t)) \equiv f(\omega) = \int_{-\infty}^{\infty} e^{i\omega t} f(t)dt = \int_{-\infty}^{\infty} e^{i\frac{\epsilon}{\hbar}t} f(t)dt = f(\epsilon) \tag{S.17}$$

$$\mathcal{F}(f(\omega)) \equiv f(t) = \frac{1}{2\pi}\int_{-\infty}^{\infty} e^{-i\omega t} f(\omega)d\omega = \frac{1}{2\pi\hbar}\int_{-\infty}^{\infty} e^{-i\frac{\epsilon}{\hbar}t} f(\epsilon)d\epsilon. \tag{S.18}$$

Equation (S.18) implies that: $\mathcal{F}(i\hbar\dot{f}(t)) = \epsilon f(\epsilon)$. We define $[A,B]_{\pm} \equiv AB \pm BA$. From this definition we define the anti-commutator: $\{A,B\} \equiv [A,B]_{+}$ and the commutator: $[A,B] \equiv [A,B]_{-}$.

### 2.2 Green's Function

The Green's function in the time domain is defined as the time ordered product of a creation and annihilation operator:

$$G_{\alpha s,\beta s'}(t-t') = -\frac{i}{\hbar}\langle T\{c_{\alpha s}(t)c^{\dagger}_{\beta s'}(t')\}\rangle. \tag{S.19}$$

Here $c^{\dagger}_{\alpha s}$ and $c_{\alpha s}$ are the creation and annihilation operators respectively, the Greek indices $\alpha, \beta$ label sites and roman labels $s, s'$ label spin. Throughout this chapter we will only consider fermionic operators these satify anti-commutation relations:

$$\{c_{\alpha s}, c^{\dagger}_{\beta s'}\} = \delta_{\alpha\beta}\delta_{ss'} \tag{S.20}$$

$$\{c_{\alpha s}, c_{\beta s'}\} = 0 = \{c^{\dagger}_{\alpha s}, c^{\dagger}_{\beta s'}\} \tag{S.21}$$

We can write the Green's function as:

$$G(t-t') = \Theta(t-t')G^{>}(t-t') + \Theta(t-t')G^{<}(t-t'). \tag{S.22}$$

Here $G^{>}(t-t')$ and $G^{<}(t-t')$ are the greater and lesser green's function respectively and $\Theta(x)$ is the Heaviside step function. Here greater and lesser refer to the time difference $t-t'$ being greater or lesser than zero. We identify:

$$G^{>}_{\alpha s,\beta s'}(t-t') = -\frac{i}{\hbar}\langle c_{\alpha s}(t) c^{\dagger}_{\beta s'}(t')\rangle, \tag{S.23}$$

$$G^{<}_{\alpha s,\beta s'}(t-t') = \frac{i}{\hbar}\langle c^{\dagger}_{\beta s'}(t') c_{\alpha s}(t)\rangle \tag{S.24}$$

We will work with retarded and advanced Green's functions respectively:

$$G^{+}_{\alpha s,\beta s'}(t-t') = -\frac{i}{\hbar}\Theta(t-t')\langle\{c_{\alpha s}(t), c^{\dagger}_{\beta s'}(t')\}\rangle, \tag{S.25}$$

$$G^{-}_{\alpha s,\beta s'}(t-t') = \frac{i}{\hbar}\Theta(t'-t)\langle\{c_{\alpha s}(t), c^{\dagger}_{\beta s'}(t')\}\rangle. \tag{S.26}$$

Here retarded and advanced refer to an event happening at time $t$ that is before time $t'$ ($t > t'$) or after time $t'$ respectively ($t < t'$). In the Fourier domain it can be shown that: $\boldsymbol{G}^{-}(\epsilon) = (\boldsymbol{G}^{+}(\epsilon))^{\dagger}$. The Green's functions in eqs. (S.23) - (S.26) are by their definition related as : $\boldsymbol{G}^{+} - \boldsymbol{G}^{-} = \boldsymbol{G}^{>} - \boldsymbol{G}^{<}$.

## 2.3 Equation of Motion Technique

In the equation of motion technique we try to get a closed expression for the retarded,advanced Green's function. We start from the definitions of the retarded Green's function (S.25) and take the derivative with respect to $t$:

$$\begin{aligned} i\hbar\dot{G}^{+}_{\kappa s'',\chi s'''}(t-t') &= \delta(t-t')\langle\{c_{\kappa s''}(t), c^{\dagger}_{\chi s'''}(t')\}\rangle \\ &+ \Theta(t-t')\langle\{\dot{c}_{\kappa s''}(t), c^{\dagger}_{\chi s'''}(t')\}\rangle. \end{aligned} \tag{S.27}$$

This equation is central to the equation of motion technique. In the equation above $\dot{c}_{\kappa s''}(t)$ appears. We can calculate the time derivative in the Heisenberg picture. In that picture the operators evolve in time and the eigenstates are stationary. The time derivative works on an operator $\boldsymbol{A}$ as:

$$i\hbar\dot{\boldsymbol{A}}(t) = [\boldsymbol{A}(t), \boldsymbol{H}]. \tag{S.28}$$

with $\boldsymbol{H}$ the Hamiltonian of the system. Given a specific Hamiltonian we can calculate $\dot{c}_{\kappa s''}(t)$ in eq. (S.27) via eq. (S.28).

## 2.4 Coupled, Interacting System

In this section we define our Hamiltonian. For a molecule that is coupled to leads and has onsite Coulomb interactions between opposite spins, the Hamiltonian is given by:

$$\begin{aligned} \boldsymbol{H} &= \sum_{\alpha s}\epsilon_{\alpha s} n_{\alpha s} + \frac{U}{2}\sum_{\alpha s} n_{\alpha s} n_{\alpha \bar{s}} \\ &+ \sum_{\alpha,\beta,s,\alpha\neq\beta} t_{\alpha\beta} c^{\dagger}_{\alpha s} c_{\beta s} + \sum_{\alpha,\beta,s,s',\alpha\neq\beta} v_{\alpha\beta,ss'} c^{\dagger}_{\alpha,s} c_{\beta,s'} \\ &+ \sum_{K}\sum_{\alpha,\beta,s,s'} V^{K}_{\alpha s',\beta s} c^{\dagger}_{\alpha s'} d^{K}_{\beta s} + V^{\dagger K}_{\alpha s',\beta s} d^{\dagger K}_{\beta s} c_{\alpha s'} + \sum_{K,\beta,s}\epsilon^{K}_{\beta,s} d^{\dagger K}_{\beta s} d^{K}_{\beta s} \end{aligned} \tag{S.29}$$

Here $c^{\dagger}_{\alpha s}$ and $c_{\alpha s}$ are the creation and annihilation operators on the molecule respectively. The onsite Hamiltonian is given by: $\boldsymbol{H}_{\text{os}} = \sum_{\alpha s} \epsilon_{\alpha s} n_{\alpha s}$ with $\epsilon_{\alpha s}$ the onsite energy for spin $s$ and $n_{\alpha s} = c^{\dagger}_{\alpha s} c_{\alpha s}$ the number operator. The Coulomb interaction gives rise to onsite interactions between opposite spins which is described by the Hamiltonian $\boldsymbol{H}_U = \frac{U}{2} \sum_{\alpha s} n_{\alpha s} n_{\alpha \bar{s}}$ here $\bar{s}$ indicates that spin $s$ is flipped and $U$ is the Coulomb interactions strength. The spin-independent hopping Hamiltonian is given by $\boldsymbol{H}_{\text{T}} = \sum_{\alpha,\beta,s,\alpha\neq\beta} t_{\alpha\beta} c^{\dagger}_{\alpha s} c_{\beta s}$ where $t_{\alpha\beta}$ describes hopping between different sites. For purely real NN hopping we have that: $t_{\alpha\beta} = t(\delta_{\alpha,\beta+1}+\delta_{\beta,\alpha+1})$. The spin-orbit coupling gives rise to a spin-dependent hopping $\boldsymbol{H}_{\text{SOC}} = \sum_{\alpha,\beta,s,s',\alpha\neq\beta} v_{\alpha\beta,ss'} c^{\dagger}_{\alpha,s} c_{\beta,s'}$. The operators $d^{\dagger K}_{\beta s}$ and $d^{K}_{\beta s}$ are the creation and annihilation operators of electrons in lead $K$ respectively. The term $\boldsymbol{H}_{\text{lead-molecule}} = \sum_K \sum_{\alpha,\beta,s,s'} V^{K}_{\alpha s',\beta s} c^{\dagger}_{\alpha s'} d^{K}_{\beta s} + h.c.$ describes the coupling between electrons on the different leads and on the molecule and $\boldsymbol{H}_{\text{lead}} = \sum_{\beta,s} \epsilon^{K}_{\beta s} d^{\dagger K}_{\beta s} d^{K}_{\beta s}$ is the Hamiltonian of the lead $K$ and $\epsilon^{K}_{\beta s}$ its onsite energy. The Hamiltonian of the molecule without interactions ($U = 0$) is defined as: $\boldsymbol{H}_0 = \boldsymbol{H}_{\text{os}} + \boldsymbol{H}_{\text{T}} + \boldsymbol{H}_{\text{SOC}}$.

## 2.5 Retarded Green's Function: Equation of Motion

Now that we have defined our Hamiltonian we first compute time-derivatives of the annihilation operators in the molecule and on the lead $\dot{c}_{\kappa s''}, \dot{d}^{K}_{\kappa s''}$ via (S.29) and (S.28), then we will obtain the equation of motion for the Green's functions.

*Time - derivatives of operators*

i) For electrons in the molecule we have:

$$\begin{aligned} i\hbar \dot{c}_{\kappa s''} &= [c_{\kappa s''}, \boldsymbol{H}] \\ &= \Big[\epsilon_{\kappa s''} + U n_{\kappa \bar{s}''}\Big] c_{\kappa s''} + \sum_{\beta} t_{\kappa\beta} c_{\beta s''} + \sum_{\beta,s} v_{\kappa\beta,s''s} c_{\beta,s} + \sum_{K,\beta s} V^{K}_{\kappa s'',\beta s} d^{K}_{\beta s} \end{aligned} \tag{S.30}$$

ii) The time derivative of the operators in the lead are given by:

$$i\hbar \dot{d}^{K}_{\kappa s''} = [d^{K}_{\kappa s''}, \boldsymbol{H}] = \epsilon^{K}_{\kappa s''} d^{K}_{\kappa s''} + \sum_{\alpha,s'} (V^{\dagger K}_{\alpha s',\kappa s''}) c_{\alpha s'} \tag{S.31}$$

*Green's Functions*

In the equation of motion of the retarded Green's function (eq. (S.27)) we replace $\dot{c}_{\kappa s''}$ by eq. (S.30) and we Fourier transform on both sides with respect to $t - t'$. This results in the following expression:

$$\begin{aligned} &\Big[\epsilon - \epsilon_{\kappa s''}\Big] G^{+}_{\kappa s'',\chi s'''}(\epsilon) - \sum_{K,\beta s} V^{K}_{\kappa s'',\beta s} G^{+,K}_{\beta s,\chi s'''}(\epsilon) - \sum_{\beta} t_{\kappa\beta} G^{+}_{\beta s'',\chi s'''}(\epsilon) - \sum_{\beta s} v_{\kappa\beta,s''s} G^{+}_{\beta s,\chi s'''}(\epsilon) \\ &= \delta_{\kappa\chi} \delta_{s''' s''} + U G^{+2}_{\kappa\bar{s}'',\kappa s'',\chi s'''}(\epsilon), \end{aligned} \tag{S.32}$$

here we define:

$$G^{+2}_{\kappa\bar{s}'',\kappa s'',\chi s'''}(t-t') = -\frac{i}{\hbar}\Theta(t-t')\langle\{n_{\kappa\bar{s}''}(t)c_{\kappa s''}(t), c^{\dagger}_{\chi s'''}(t')\}\rangle, \tag{S.33}$$

$$G^{+,K}_{ab,cd}(t-t') = -\frac{i}{\hbar}\Theta(t-t')\langle\{d^{K}_{ab}(t), c^{\dagger}_{cd}(t')\}\rangle \tag{S.34}$$

with $G^{+2}_{\kappa\bar{s}'',\kappa s'',\chi s'''}(\epsilon)$ and $G^{+,K}_{ab,cd}(\epsilon)$ the respective Fourier transforms. To solve for $G^{+,K}_{ab,cd}(\epsilon)$ we first take a derivative with respect to $t$ of eq. (S.34) and use eq. (S.31) for $\dot{d}^{K}_{ab}(t)$, then we Fourier transform and solve to to find: $G^{+,K}_{ab,cd}(\epsilon) = \sum_{ef} \frac{V^{K*}_{ef,ab}}{(\epsilon-\epsilon^{K}_{ab})} G^{+}_{ef,cd}(\epsilon)$. We then use this to obtain:

$$\sum_{K,\beta s} V^{K}_{\kappa s'',\beta s} G^{+,K}_{\beta s,\chi s'''}(\epsilon) = \sum_{K,ef}\Big(\sum_{\beta s}\frac{V^{K}_{\kappa s''\beta s}V^{K*}_{ef,\beta s}}{\epsilon-\epsilon^{K}_{\beta s}}\Big)G^{+}_{ef,\chi s'''}(\epsilon) = \sum_{K,ef}\Sigma^{K}_{\kappa s'',ef}G^{+}_{ef,\chi s'''}(\epsilon). \tag{S.35}$$

Where we define:

$$\Sigma^{K}_{ab,cd}(\epsilon) = \sum_{\chi\beta}\frac{V^{K}_{ab,\chi\beta}V^{K*}_{cd,\chi\beta}}{\epsilon-\epsilon^{K}_{\chi\beta}} \tag{S.36}$$

as the retarded self-energy of lead $K$. It is common to split the retarded self energy into an imaginary and a real part:

$$\Sigma^{K}_{ab,cd}(\epsilon) = \Lambda_{ab,cd}(\epsilon) - \frac{i}{2}\Gamma_{ab,cd}(\epsilon), \tag{S.37}$$

where $\Lambda_{ab,cd}(\epsilon)$ and $\Gamma_{ab,cd}(\epsilon)$ are real numbers. Now eq. (S.32) becomes:

$$\begin{aligned}&\Big[\epsilon-\epsilon_{\kappa s''}\Big]G^{+}_{\kappa s'',\chi s'''}(\epsilon) - \sum_{K,ef}\Sigma^{K}_{\kappa s'',ef}G^{+}_{ef',\chi s'''}(\epsilon) - \sum_{\beta}t_{\kappa\beta}G^{+}_{\beta s'',\chi s'''}(\epsilon) - \sum_{\beta s}v_{\kappa\beta,s''s}G^{+}_{\beta s,\chi s'''}(\epsilon)\\ &= \delta_{\kappa\chi}\delta_{s'''s''} + UG^{+2}_{\kappa\bar{s}'',\kappa s'',\chi s'''}(\epsilon).\end{aligned} \tag{S.38}$$

## 2.6 Hartree - Fock Approximation

Normally in the Hartree-Fock approximation the second order and first order Greens function are related as:

$$G^{+2}_{\kappa\bar{s}'',\kappa s'',\chi s'''}(\epsilon) = \langle n_{\kappa\bar{s}''}\rangle G^{+}_{\kappa s'',\chi s'''}(\epsilon). \tag{S.39}$$

We do this to cut of the hierarchy of the Green's function. Physically it means that you neglect correlations between all occupation numbers. Equation (S.39) is the result of considering the Wick contraction of the first two equal time operators: $c^{\dagger}_{\kappa\bar{s}''}c_{\kappa\bar{s}''}c_{\kappa s''}$ in eq. (S.33). However we can also consider the contraction of the first with the third (note that $\langle c_{ab}c_{ef}\rangle = 0$) resulting in:

$$c^{\dagger}_{\kappa\bar{s}''}c_{\kappa\bar{s}''}c_{\kappa s''} = \langle n_{\kappa\bar{s}''}\rangle c_{\kappa s''} - \langle c^{\dagger}_{\kappa\bar{s}''}c_{\kappa s''}\rangle c_{\kappa\bar{s}''}. \tag{S.40}$$

The expectation value $\langle c^\dagger_{\kappa\bar{s}''} c_{\kappa s''}\rangle$ is nonzero in the presence of spin-orbit coupling (but vanishes if the spin-orbit coupling is set to zero). Thus we can approximate the second order Green's function as:

$$G^{+2}_{\kappa\bar{s}'',\kappa s'',\chi s'''}(\epsilon) = \langle n_{\kappa\bar{s}''}\rangle G^{+}_{\kappa s'',\chi s'''}(\epsilon) - \langle c^\dagger_{\kappa\bar{s}''} c_{\kappa s''}\rangle G^{+}_{\kappa\bar{s}'',\chi s'''}(\epsilon). \tag{S.41}$$

for a system with spin-orbit coupling. We now substitute eq. (S.41) into eq. (S.38) to find the equation of motion:

$$\begin{aligned}&\Big[\epsilon - \epsilon_{\kappa s''}\Big] G^{+}_{\kappa s'',\chi s'''}(\epsilon) - U\langle n_{\kappa\bar{s}''}\rangle G^{+}_{\kappa s'',\chi s'''}(\epsilon) + U\langle c^\dagger_{\kappa\bar{s}''} c_{\kappa s''}\rangle G^{+}_{\kappa\bar{s}'',\chi s'''}(\epsilon)\\ &+ \sum_{K,e,f} \Sigma^{K}_{\kappa s'',ef} G^{+}_{ef,\chi s'''}(\epsilon) - \sum_{\beta} t_{\kappa\beta s''} G^{+}_{\beta s',\chi s'''}(\epsilon) - \sum_{\beta s} v_{\kappa\beta s'' s} G^{+}_{\beta s,\chi s'''}(\epsilon) = \delta_{\kappa\chi}\delta_{s''' s''}\end{aligned} \tag{S.42}$$

We can now solve this equation for $\mathbf{G}^{+}$. The retarded Green's function in matrix notation is given by:

$$\mathbf{G}^{+}_{\text{HFA}}(\epsilon) = \Big[\epsilon\mathbf{1} - \boldsymbol{H}_0 - U\boldsymbol{n} + U\boldsymbol{\rho} - \boldsymbol{\Sigma}\Big]^{-1}. \tag{S.43}$$

Here $\boldsymbol{\Sigma} = \sum_K \boldsymbol{\Sigma}^K$ is the sum of all retarded self-energies, $\boldsymbol{n} = \sum_{\kappa s}\langle n_{\kappa\bar{s}}\rangle n_{\kappa s}$ is a diagonal matrix with the electron densities on the diagonal and $\boldsymbol{\rho} = \sum_{\kappa s}\langle c^\dagger_{\kappa\bar{s}} c_{\kappa s}\rangle c^\dagger_{\kappa s} c_{\kappa\bar{s}}$ is an off-diagonal matrix.
The term $\boldsymbol{\rho}$ describes hopping between up and down electrons on the same site, therefore it can have an imaginary and a real part: $\langle c^\dagger_{\kappa\bar{s}''} c_{\kappa s''}\rangle = a + ib$ and its complex conjugate $a - ib$ which makes that $\boldsymbol{\rho}$ is proportional to the Pauli matrices: $a\sigma_x + b\sigma_y$. Thus we can interpret this term as an onsite spin in the $x$ and $y$ direction. That is why some references call this the non-collinear Hubbard model [2], since the spin is not parallel to $z$ direction. Therefore we call the Green's function in eq. (S.43) the non-collinear Hartree Fock Green's function.

The Hartree Fock Green's function is usually made particle-hole symmetric by subtracting a factor of 1/2 from the electron densities: $U\Big(\langle n_{\kappa\bar{s}}\rangle - \frac{1}{2}\Big) n_{\kappa s}$. For a bipartite lattice the density of states are symmetric around the energy $E = 0$. When the factor of 1/2 is omitted the density of states are symmetric around the energy $E = \frac{U}{2}$.

## 2.7 Hubbard One Approximation

In the Hubbard One approximation we solve for $G^{+2}_{\kappa\bar{s}'',\kappa s'',\chi s'''}(\epsilon)$ instead of approximating it (via eq. (S.41) ). A solution is sought via the equation of motion of $G^{+2}_{\kappa\bar{s}'',\kappa s'',\chi s'''}(\epsilon)$, which is obtained by taking the time derivative of eq. (S.33) with respect to $t$:

$$\begin{aligned}\dot{G}^{+2}_{\kappa\bar{s}'',\kappa s'',\chi s'''}(t-t') = &-\frac{i}{\hbar}\delta(t-t')\langle\{n_{\kappa\bar{s}''}(t)c_{\kappa s''}(t), c^\dagger_{\chi s'''}(t')\}\rangle\\ &-\frac{i}{\hbar}\Theta(t-t')\langle\{\dot{n}_{\kappa\bar{s}''}(t)c_{\kappa s''}(t), c^\dagger_{\chi s'''}(t')\}\rangle.\\ &-\frac{i}{\hbar}\Theta(t-t')\langle\{n_{\kappa\bar{s}''}(t)\dot{c}_{\kappa s''}(t), c^\dagger_{\chi s'''}(t')\}\rangle.\end{aligned} \tag{S.44}$$

In eq. (S.44) the time derivative of the number operator $\dot{n}_{\kappa\bar{s}''}(t)$ is given by:

$$
\begin{aligned}
\dot{n}_{\kappa\bar{s}''}(t) &= [n_{\kappa\bar{s}''}, \boldsymbol{H}] \\
&= \sum_{\beta} t_{\kappa\beta} c^{\dagger}_{\kappa\bar{s}''} c_{\beta\bar{s}''} - t_{\beta\kappa} c^{\dagger}_{\beta\bar{s}''} c_{\kappa\bar{s}''} \\
&+ \sum_{\beta,s'} v_{\kappa\beta,\bar{s}''s'} c^{\dagger}_{\kappa\bar{s}''} c_{\beta s'} - v_{\beta\kappa,s'\bar{s}''} c^{\dagger}_{\beta s'} c_{\kappa\bar{s}''} \\
&+ \sum_{K} \sum_{\beta,s} V^{K}_{\kappa\bar{s}'',\beta s} c^{\dagger}_{\kappa\bar{s}''} d^{K}_{\beta s} - V^{\dagger K}_{\kappa\bar{s}'',\beta s} d^{\dagger K}_{\beta s} c_{\kappa\bar{s}''}. 
\end{aligned} \tag{S.45}
$$

In the original work of Hubbard[3] the time derivative of the number operator is set to zero ($\dot{n}_{\kappa\bar{s}''}(t) = 0$). We will not put that into eq. (S.44) directly (for reasons that will become clear later). In eq. (S.44) we substitute eqs. (S.30) for $\dot{c}_{\kappa s''}(t)$ and eq. (S.45) for $\dot{n}_{\kappa\bar{s}''}(t)$ and Fourier transform the resulting equation with respect to $t - t'$:

$$
\begin{aligned}
(\epsilon - \epsilon_{\kappa s''}) G^{+2}_{\kappa\bar{s}'',\kappa s'',\chi s'''} &= \delta_{\kappa\chi}\delta_{s''s'''}\langle n_{\kappa\bar{s}''}\rangle - \delta_{\kappa\chi}\delta_{\bar{s}''s'''}\langle c^{\dagger}_{\kappa\bar{s}''} c_{\kappa s''}\rangle \\
&+ U G^{+3}_{\kappa s''\chi s'''}(\epsilon) \\
&+ \sum_{\beta} t_{\kappa\beta} G^{+2}_{\kappa\bar{s}'',\beta s'',\chi s'''} + \sum_{\beta s} v_{\kappa\beta,ss''} G^{+2}_{\kappa\bar{s}'',\beta s,\chi s'''} \\
&+ \sum_{K,\beta s'} V^{K}_{\kappa s'',\beta s'} G^{+2,K}_{\kappa\bar{s}'',\beta s',\chi s'''} \\
&+ \sum_{\beta} t_{\kappa\beta} \overline{G}^{+2}_{\kappa\bar{s}'',\beta\bar{s}'',\kappa s'',\chi s'''} - t_{\beta\kappa} \overline{G}^{+2}_{\beta\bar{s}'',\kappa\bar{s}'',\kappa s'',\chi s'''} \\
&+ \sum_{\beta,s'} v_{\kappa\beta,\bar{s}''s'} \overline{G}^{+2}_{\kappa\bar{s}'',\beta s',\kappa s'',\chi s'''} - v_{\beta\kappa,s'\bar{s}''} \overline{G}^{+2}_{\beta s',\kappa\bar{s}'',\kappa s'',\chi s'''} \\
&+ \sum_{K} \sum_{\beta,s} V^{K}_{\kappa\bar{s}'',\beta s} \overline{G}^{+2,K}_{\kappa\bar{s}'',\beta s,\kappa s'',\chi s'''} - (V^{K}_{\kappa\bar{s}'',\beta s})^{\dagger} \overline{G}^{-2,K}_{\beta s,\kappa\bar{s}'',\kappa s'',\chi s'''}. 
\end{aligned} \tag{S.46}
$$

Note the appearance of $\langle c^{\dagger}_{\kappa\bar{s}''} c_{\kappa s''}\rangle$ which usually is set to zero[3,4] but not here (due to spin-orbit coupling). Furthermore we define:

$$
G^{+2}_{\kappa s'',ef,\chi s'''}(t - t') = -\frac{i}{\hbar}\Theta(t - t')\langle\{n_{\kappa\bar{s}''}(t) c_{ef}(t), c^{\dagger}_{\chi s'''}(t')\}\rangle \tag{S.47}
$$

$$
G^{+2,K}_{\kappa\bar{s}'',\beta s',\chi s'''}(t - t') = -\frac{i}{\hbar}\Theta(t - t')\langle\{n_{\kappa\bar{s}''}(t) d^{K}_{\beta s'}(t), c^{\dagger}_{\chi s'''}(t')\}\rangle \tag{S.48}
$$

$$
\overline{G}^{+2}_{ab,cd,ef,gh}(t - t') = -\frac{i}{\hbar}\Theta(t - t')\langle\{c^{\dagger}_{ab}(t) c_{cd}(t) c_{ef}(t), c^{\dagger}_{gh}(t')\}\rangle \tag{S.49}
$$

$$
\overline{G}^{+2,K}_{ab,cd,ef,gh}(t - t') = -\frac{i}{\hbar}\Theta(t - t')\langle\{c^{\dagger}_{ab}(t) d^{K}_{cd}(t) c_{ef}(t), c^{\dagger}_{gh}(t')\}\rangle \tag{S.50}
$$

$$
\overline{G}^{-2,K}_{ab,cd,ef,gh}(t - t') = -\frac{i}{\hbar}\Theta(t - t')\langle\{d^{\dagger K}_{ab}(t) c_{cd}(t) c_{ef}(t), c^{\dagger}_{gh}(t')\}\rangle \tag{S.51}
$$

with $G^{+2}_{\kappa\bar{s}'',ef,\chi s'''}(\epsilon)$ , $G^{+2,K}_{\kappa\bar{s}'',\beta s',\chi s'''}(\epsilon)$,$\overline{G}^{+2}_{ab,cd,ef,gh}(\epsilon)$ , $\overline{G}^{+2,K}_{ab,cd,ef,gh}(\epsilon)$ and $\overline{G}^{-2,K}_{ab,cd,ef,gh}(\epsilon)$ as the respective Fourier transforms. The third order Green's function is defined as:

$$
G^{3+}_{\kappa s'',\chi s'''}(t - t') = -\frac{i}{\hbar}\Theta(t - t')\langle\{n_{\kappa\bar{s}''}(t) n_{\kappa\bar{s}''}(t) c_{\kappa s''}(t), c(t')^{\dagger}_{\chi s'''}\}\rangle, \tag{S.52}
$$

and $G^{3+}_{\kappa s'',\chi s'''}(\epsilon)$ its Fourier transform. For fermions working on a number state we have that $n^2_{\kappa\bar{s}''} = n_{\kappa\bar{s}''}$, thus we conclude $G^{3+}_{\kappa s'',\chi s'''}(\epsilon) = G^{+2}_{\kappa\bar{s}'',\kappa s'',\chi s'''}(\epsilon)$. Now in order to solve eq. S.46 in terms of $G^{+2}_{\kappa\bar{s}'',\kappa s'',\chi s'''}$ we need to approximate.

*Approximations*

As an approximation we will consider all non-zero possible Wick contractions of the equal time operators in eqs. (S.47) -(S.51):

$$G^{+2}_{\kappa\bar{s}'',\alpha s,\chi s'''} = \langle n_{\kappa\bar{s}''}\rangle G^{+}_{\alpha s,\chi s'''} - \langle c^{\dagger}_{\kappa\bar{s}''} c_{\alpha s}\rangle G^{+}_{\kappa\bar{s}'',\chi s'''} \tag{S.53}$$

$$G^{+2,K}_{\kappa\bar{s}'',\beta s',\chi s'''} = \langle n_{\kappa\bar{s}''}\rangle G^{+,K}_{\beta s',\chi s'''} - \langle c^{\dagger}_{k\bar{s}''} d^{K}_{\beta s'}\rangle G^{+}_{\kappa\bar{s}'',\chi s'''} \tag{S.54}$$

$$\overline{G}^{+2}_{ab,cd,ef,gh} = \langle c^{\dagger}_{ab} c_{cd}\rangle G^{+}_{ef,gh} - \langle c^{\dagger}_{ab} c_{ef}\rangle G^{+}_{cd,gh} \tag{S.55}$$

$$\overline{G}^{+2,K}_{ab,cd,ef,gh} = -\langle c^{\dagger}_{ab} c_{ef}\rangle G^{+,K}_{cd,gh} + \langle c^{\dagger}_{ab} d_{cd}\rangle G^{+}_{ef,gh} \tag{S.56}$$

$$\overline{G}^{-2,K}_{ab,cd,ef,gh} = \langle d^{\dagger}_{ab} c_{cd}\rangle G^{+}_{ef,gh} - \langle d^{\dagger}_{ab} c_{ef}\rangle G^{+}_{cd,gh}. \tag{S.57}$$

Putting the approximations eqs. (S.53)-(S.57) into eq. (S.46) we obtain after some rearrangement:

$$\begin{aligned}
(\epsilon - \epsilon_{\kappa s''} - U) G^{+2}_{\kappa\bar{s}'',\kappa s'',\chi s'''} &= \delta_{\kappa\chi}\delta_{s''s'''}\langle n_{\kappa\bar{s}''}\rangle - \delta_{\kappa\chi}\delta_{\bar{s}''s'''}\langle c^{\dagger}_{\kappa\bar{s}''} c_{\kappa s''}\rangle \\
&+ \langle n_{\kappa\bar{s}''}\rangle \Big( \sum_{\beta} t_{\kappa\beta} G^{+}_{\beta s'',\chi s'''} + \sum_{\beta s} v_{\kappa\beta,ss''} G^{+}_{\beta s,\chi s'''} + \sum_{K,\beta s'} V^{K}_{\kappa s'',\beta s'} G^{+,K}_{\beta s',\chi s'''} \Big) \\
&- \langle c^{\dagger}_{\kappa\bar{s}''} c_{\kappa s''}\rangle \Big( \sum_{\beta} t_{\kappa\beta} G^{+}_{\beta\bar{s}'',\chi s'''} + \sum_{\beta,s'} v_{\kappa\beta,\bar{s}''s'} G^{+}_{\beta s',\chi s'''} + \sum_{K}\sum_{\beta s} V^{K}_{\kappa\bar{s}'',\beta s} G^{K}_{\beta s,\chi s'''} \Big) \\
&+ \Big[ \sum_{\beta} t_{\beta\kappa}\langle c^{\dagger}_{\beta\bar{s}''} c_{\kappa s''}\rangle - t_{\kappa\beta}\langle c^{\dagger}_{\kappa\bar{s}''} c_{\beta s''}\rangle \\
&+ \sum_{\beta,s} v_{\beta\kappa,s\bar{s}''}\langle c^{\dagger}_{\beta s} c_{\kappa s''}\rangle - v_{\kappa\beta,ss''}\langle c^{\dagger}_{\kappa\bar{s}''} c_{\beta s}\rangle \\
&+ \sum_{K}\sum_{\beta,s} (V^{K}_{\kappa\bar{s}'',\beta s})^{\dagger}\langle d^{\dagger K}_{\beta s} c_{\kappa s''}\rangle - V^{K}_{\kappa s'',\beta s'}\langle c^{\dagger}_{k\bar{s}''} d^{K}_{\beta s'}\rangle \Big] G^{+}_{\kappa\bar{s}'',\chi s'''} \\
&+ \Big[ \sum_{\beta} t_{\kappa\beta}\langle c^{\dagger}_{\kappa\bar{s}''} c_{\beta\bar{s}''}\rangle - t_{\beta\kappa}\langle c^{\dagger}_{\beta\bar{s}''} c_{\kappa\bar{s}''}\rangle \\
&+ \sum_{\beta,s} \langle c^{\dagger}_{\kappa\bar{s}''} c_{\beta s'}\rangle v_{\kappa\beta,\bar{s}''s} - v_{\beta\kappa,s\bar{s}''}\langle c^{\dagger}_{\beta s} c_{\kappa\bar{s}''}\rangle \\
&+ \sum_{K}\sum_{\beta,s} V^{K}_{\kappa\bar{s}'',\beta s}\langle c^{\dagger}_{\kappa\bar{s}''} d^{K}_{\beta s}\rangle - V^{\dagger K}_{\kappa\bar{s}'',\beta s}\langle d^{\dagger K}_{\beta s} c_{\kappa\bar{s}''}\rangle \Big] G^{+}_{\kappa s'',\chi s'''}.
\end{aligned} \tag{S.58}$$

The term in front of $G^{+}_{\kappa s'',\chi s'''}$ in eq. (S.58) is recognized as the expectation value of the time derivative of the number operator: $\langle \dot{n}_{\kappa s''}(t)\rangle$ (via eq. (S.45)). We set this expectation value to zero since in steady state the electron density is constant. To show that the term in front of $G^{+}_{\kappa\bar{s}'',\chi s'''}$ in eq. (S.58) is zero we first calculate the time derivative of $c^{\dagger}_{\kappa\bar{s}''}(t) c_{\kappa s''}(t)$:

$$\begin{aligned}\frac{d}{dt}c^{\dagger}_{\kappa\bar{s}''}(t)c_{\kappa s''}(t) &= [c^{\dagger}_{\kappa\bar{s}''}c_{\kappa s''},\boldsymbol{H}] \\ &= \sum_{\beta} t_{\kappa\beta}c^{\dagger}_{\kappa\bar{s}''}c_{\beta s''} - t_{\beta\kappa}c^{\dagger}_{\beta\bar{s}''}c_{\kappa s''} \\ &+ \sum_{\beta s} v_{\kappa\beta,ss''}c^{\dagger}_{\kappa\bar{s}''}c_{\beta s''} - v_{\beta\kappa,s\bar{s}''}c^{\dagger}_{\beta\bar{s}''}c_{\kappa s''} \\ &+ \sum_{K,\beta s} V^{K}_{\kappa s'',\beta s}c^{\dagger}_{\kappa\bar{s}''}d^{K}_{\beta s} - V^{\dagger K}_{\kappa\bar{s}'',\beta s}d^{K}_{\beta s}c_{\kappa s''},\end{aligned} \tag{S.59}$$

where we used that $\epsilon_{\alpha s} = \epsilon_{\alpha\bar{s}}$ (no magnetic field in the molecule). With the result in eq. (S.59) we see that the term in front of $G^{+}_{\kappa\bar{s}'',\chi s'''}$ in eq. (S.58) is equal to $:-\langle\frac{d}{dt}c^{\dagger}_{\kappa\bar{s}''}c_{\kappa s''}\rangle$. We set this term to zero as well since the average spin in the $x, y$ direction will be fixed in steady state. Given these approximations we obtain the following equation of motion:

$$\begin{aligned}(\epsilon - \epsilon_{\kappa s''} - U)G^{+2}_{\kappa\bar{s}'',\kappa s'',\chi s'''} &= \langle n_{\kappa\bar{s}''}\rangle\delta_{\kappa\chi}\delta_{s'''s''} - \delta_{\kappa\chi}\delta_{\bar{s}''s'''}\langle c^{\dagger}_{\kappa\bar{s}''}c_{\kappa s''}\rangle \\ &+ \langle n_{\kappa\bar{s}''}\rangle\Big(\sum_{\beta} t_{\kappa\beta}G^{+}_{\beta s'',\chi s'''} + \sum_{\beta s} v_{\kappa\beta,ss''}G^{+}_{\beta s,\chi s'''} + \sum_{K,ef}\Sigma^{K}_{\kappa s'',ef}G^{+}_{ef,\chi s'''}\Big) \\ &- \langle c^{\dagger}_{\kappa\bar{s}''}c_{\kappa s''}\rangle\Big(\sum_{\beta} t_{\kappa\beta}G^{+}_{\beta\bar{s}'',\chi s'''} + \sum_{\beta,s'} v_{\kappa\beta,\bar{s}''s'}G^{+}_{\beta s',\chi s'''} + \sum_{K}\sum_{ef}\Sigma^{K}_{\kappa\bar{s}'',ef}G^{+}_{ef,\chi s'''}\Big)\end{aligned} \tag{S.60}$$

Where we also used eq. (S.35) and the definition of the retarded self energy (eq. (S.36)). Now we come back to reason why we did not put the approximation $\dot{n}_{\kappa\bar{s}''} = 0$ directly in (S.44). Had we done that, then the terms in front of $G^{+}_{\kappa s'',\chi s'''}$ and $G^{+}_{\kappa\bar{s}'',\chi s'''}$ could not have been recognized as $\langle\dot{n}_{\kappa s''}(t)\rangle$ and $-\langle\frac{d}{dt}c^{\dagger}_{\kappa\bar{s}''}c_{\kappa s''}\rangle$ respectively, because there would be correlators missing that otherwise would have come from $\dot{n}_{\kappa\bar{s}''}(t)$. Usually the relation: $\dot{n}_{\kappa\bar{s}''}(t) = 0$ is used directly and then (after some Wick contractions) individual correlators are set to zero. We point out that this 'usual' procedure results in the same equation of motion for $G^{+2}$ if $\langle c^{\dagger}_{\kappa\bar{s}''}c_{\kappa s''}\rangle = 0$. We now solve for the retarded Green's function by via eqs. (S.38) and (S.60) which in matrix notation become:

$$(\epsilon\boldsymbol{I} - \boldsymbol{H}_0 - \boldsymbol{\Sigma})\boldsymbol{G}^{+} - U\boldsymbol{G}^{+2} = \boldsymbol{I}, \tag{S.61}$$

and:

$$\boldsymbol{G}^{+2} = \Big[(\epsilon\boldsymbol{I} - \boldsymbol{H}_{\text{os}} - U\boldsymbol{I})\Big]^{-1}\Big[(\boldsymbol{n} - \boldsymbol{\rho})\Big(\boldsymbol{I} + (\boldsymbol{H}_{\text{T}} + \boldsymbol{H}_{\text{SOC}} + \boldsymbol{\Sigma})\boldsymbol{G}^{+}\Big)\Big] \tag{S.62}$$

respectively. Substituting (S.62) into (S.61) and solving for $\boldsymbol{G}^{+}$ gives:

$$\begin{aligned}\boldsymbol{G}^{+}_{\text{HIA}}(\epsilon) = &\frac{1}{\Big[(\epsilon\boldsymbol{I} - \boldsymbol{H}_{\text{os}} - U\boldsymbol{I})(\boldsymbol{E} - \boldsymbol{H}_{\text{os}}) - \Big(\epsilon\boldsymbol{I} - \boldsymbol{H}_{\text{os}} - U(\boldsymbol{I} - \boldsymbol{n} + \boldsymbol{\rho})\Big)\Big(\boldsymbol{H}_{\text{T}} + \boldsymbol{H}_{\text{SOC}} + \boldsymbol{\Sigma}\Big)\Big]} \\ &\times\Big[\epsilon\boldsymbol{I} - \boldsymbol{H}_{\text{os}} - U(\boldsymbol{I} - \boldsymbol{n} + \boldsymbol{\rho})\Big].\end{aligned} \tag{S.63}$$

Which we will call the non-collinear Hubbard One Green's function. If the spin-orbit coupling is small we can set $\boldsymbol{\rho} = 0$ and the Green's function reduces to its well-known form [4].

Now follow some sanity checks. It is common to write the lesser, greater Green's function in the Fourier domain as:

$$\boldsymbol{G}^{<} = \boldsymbol{G}^{+}\Big[\sum_{K}\boldsymbol{\Gamma}^{K} f_{K}\Big]\boldsymbol{G}^{-}, \quad \boldsymbol{G}^{>} = \boldsymbol{G}^{+}\Big[\sum_{K}\boldsymbol{\Gamma}^{K}(1-f_{K})\Big]\boldsymbol{G}^{-}, \tag{S.64}$$

with $\boldsymbol{\Gamma}^{K} = 2\mathrm{Im}[(\boldsymbol{\Sigma}^{K})^{\dagger}]$ and $f_K$ the fermi-dirac distribution of lead $K$. This implies that $\boldsymbol{G}^{>} - \boldsymbol{G}^{<} = i\boldsymbol{G}^{+}\boldsymbol{\Gamma}\boldsymbol{G}^{-}$. By definition of the Green's function we should then find $\boldsymbol{G}^{>} - \boldsymbol{G}^{<} \equiv \boldsymbol{G}^{+} - \boldsymbol{G}^{-} = i\boldsymbol{G}^{+}\sum_{K}\boldsymbol{\Gamma}^{K}\boldsymbol{G}^{-}$. To show that this we first rewrite the Green's function as:

$$\boldsymbol{G}^{+} = [\boldsymbol{g}_0^{-1} - \boldsymbol{\Sigma}]^{-1}, \tag{S.65}$$

with $\boldsymbol{g}_0^{-1} = \Big[\epsilon\boldsymbol{I} - \boldsymbol{H}_{\mathrm{os}} - U(\boldsymbol{I} - \boldsymbol{n} + \boldsymbol{\rho})\Big]^{-1}\Big[(\epsilon\boldsymbol{I} - \boldsymbol{H}_{\mathrm{os}} - U\boldsymbol{I})(\boldsymbol{E} - \boldsymbol{H}_{\mathrm{os}})\Big] - (\boldsymbol{H}_{\mathrm{T}} + \boldsymbol{H}_{\mathrm{SOC}})$. From the fact that $\epsilon\boldsymbol{I}, U\boldsymbol{I}, \boldsymbol{H}_{\mathrm{os}}$ are diagonal and $\boldsymbol{H}_{\mathrm{T}}, \boldsymbol{H}_{\mathrm{SOC}}$ are hermitian it follows that $\boldsymbol{g}_0^{-1} = (\boldsymbol{g}_0^{-1})^{\dagger}$. From this we can easily verify that: $\boldsymbol{G}^{+} - \boldsymbol{G}^{-} = i\boldsymbol{G}^{+}\sum_{K}\boldsymbol{\Gamma}^{K}\boldsymbol{G}^{-}$ is satisfied. For small $U$ we should retrieve the Hartree-Fock Green's function. We can expand $\boldsymbol{g}_0^{-1}$ to first order in $U$:

$$\boldsymbol{g}_0^{-1} = \boldsymbol{g}_0^{-1}|_{U=0} + U\partial_U \boldsymbol{g}_0^{-1}{}_{U=0} = \boldsymbol{E} - \boldsymbol{H}_{\mathrm{os}} - U(\boldsymbol{n} - \boldsymbol{\rho})_{U=0} - \boldsymbol{H}_{\mathrm{T}} - \boldsymbol{H}_{\mathrm{SOC}} \tag{S.66}$$

Here we used $\frac{d\boldsymbol{A}^{-1}}{dx} = \boldsymbol{A}^{-1}\frac{d\boldsymbol{A}}{dx}\boldsymbol{A}^{-1}$ .Plugging the equation above back into eq. (S.65) $\boldsymbol{G}^{+} = \Big[\boldsymbol{E} - \boldsymbol{H}_{\mathrm{os}} - U(\boldsymbol{n} - \boldsymbol{\rho})_{U=0} - \boldsymbol{H}_{\mathrm{T}} - \boldsymbol{H}_{\mathrm{SOC}} - \boldsymbol{\Sigma}\Big]^{-1}$ which is identical to the non collinear Hartree Fock Green's function (eq. (S.43)) when $\boldsymbol{n}, \boldsymbol{\rho}$ are expanded to zeroth order in $U$. These two sanity checks support that the Green's function found eq. (S.63) is correct.

# 3 Evenness or Oddness of Magnetocurrent

In this section we will present the results regarding the odd or even behaviour of $\Delta I(m, V)$. In order to quantify the degree to which a function is odd or even, we define the quantity $P(V)$:

$$P(V) = \frac{\int_0^V O[\Delta I(m, V')]^2 - E[\Delta I(m, V')]^2 dV'}{\int_0^V O[\Delta I(m, V')]^2 + E[\Delta I(m, V')]^2 dV'}. \tag{S.67}$$

Here $O[\Delta I(m, V')], E[\Delta I(m, V')]$ are the odd and even part of $\Delta I(m, V')$ in bias voltage respectively. For functions that are purely odd or even we have $P(V) = 1$ and $P(V) = -1$ respectively. If $P(V) > 1/4$ we say that $\Delta I(m, V)$ is odd, if $P(V) < -1/4$, $\Delta I(m, V)$ is even and if $|P(y)| \leq \frac{1}{4}$, then we say that $\Delta I(m, V)$ is neither odd nor even. For small $V$ the errors on $\Delta I(m, V)$ are relatively large, therefore $P(V)$ is sensitive to these errors for small bias. Therefore we neglect $|\Delta I(m, V)| < 10^{-8}$. In fig. S1 we see the $P(V)$ parameter for the helical geometry in the HFA and HIA approximation. In fig. S1a $P(V)$ is plotted for $E_{\mathrm{F}} \neq \frac{U}{2}$ in the HFA. For $\frac{U}{t} = 0.1$ and for $\frac{U}{t} = 2$ the magnetocurrent is even for small bias and odd for large bias respectively. In fig. S1b) $P(V)$ is plotted for $E_{\mathrm{F}} \neq \frac{U}{2}$ in the HIA. Here the magnetocurrent is and odd function. The unclear symmetry of the magnetocurrent for $U/t = \frac{1}{2}$ and small bias we attribute to the relatively large numerical errors present in that range (especially for $\lambda = 10^{-3}$ where the signal is very weak). Since both $U/t, \lambda/t$ are small the magnetocurrent is expected to be small and its behaviour sensitive to numerical errors. In fig. S1c $P(V)$ is plotted for

$E_{\rm F} = \frac{U}{2}$ in the HFA and $\Delta I(m,V)$ is perfectly odd function of bias voltage for small $U/t$ and dominantly odd voor large $U/t$. In fig. S1d) $P(V)$ is plotted for $E_{\rm F} = \frac{U}{2}$ in the HIA. We see that $\Delta I(m,V)$ is a pure odd function of bias voltage for large $U/t$ and a dominantly odd function for small $U/t$. From fig. S1 we can conclude that the magnetocurrent is an odd function of bias voltage.

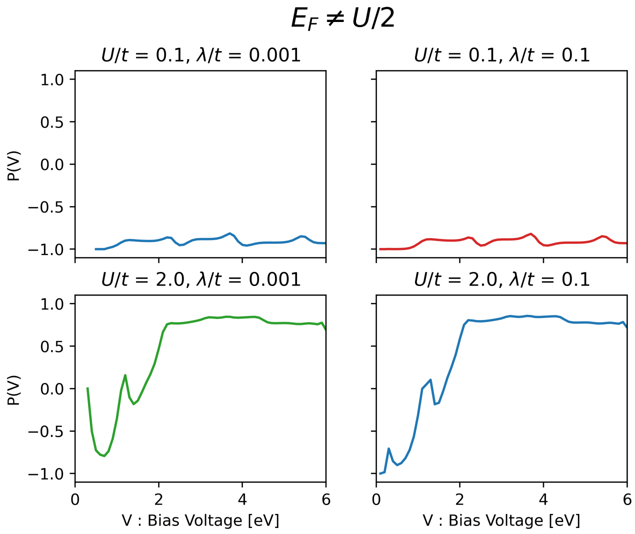


(a) HFA

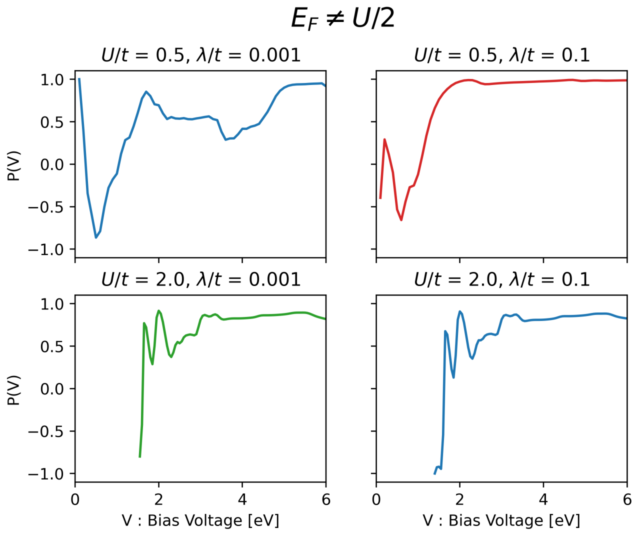


(b) HIA

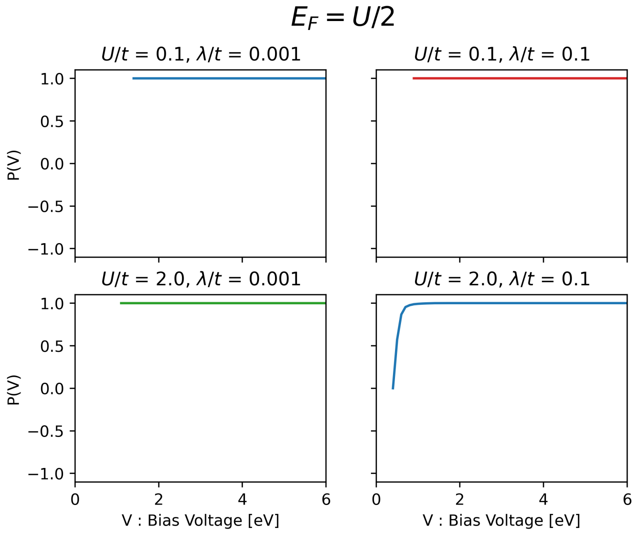


(c) HFA

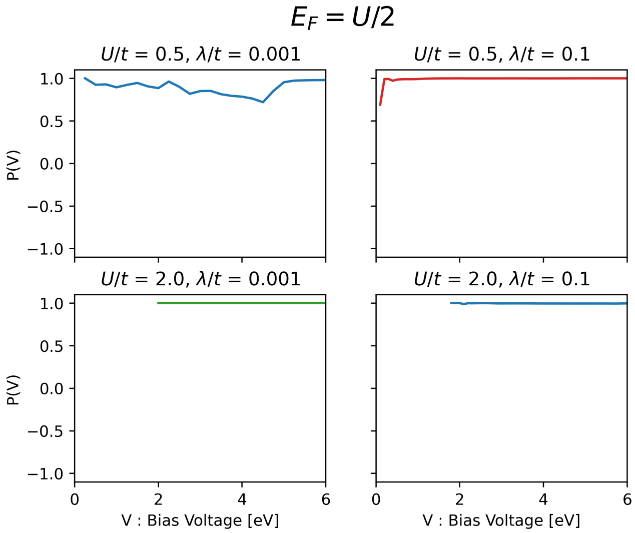


(d) HIA

Figure S1: $P(V)$ for the the helical geometry. Asymmetric chemical potential **(a)** in the HFA, **(b)** in the HIA. Symmetric chemical potential: **(c)** in the HFA, **(d)** in the HIA.

# 4 Bibliography

## References

(1) Jacquet, P. A. ThermoElectric Transport Properties of a Chain of Quantum Dots with Self-Consistent Reservoirs. *Journal of Statistical Physics* **2009**, *134*, 709–748.

(2) Pasrija, K.; Kumar, S. Noncollinear and noncoplanar magnetic order in the extended Hubbard model on anisotropic triangular lattice. *Physical Review B* **2016**, *93*, DOI: 10.1103/physrevb.93.195110.

(3) Hubbard, J. Electron Correlations in Narrow Energy Bands. *Proceedings of the Royal Society of London. Series A, Mathematical and Physical Sciences* **1963**, *276*, 238–257.

(4) Haug, H. J. W.; Jauho, A. P., *Quantum Kinetics in Transport and Optics of Semiconductors*; Springer: 2008.
